# Examining contraception-related discourse on social media after the *Dobbs v. Jackson Women’s Health Organization* Supreme Court decision: a textual analysis of user-generated content on X (formerly Twitter)

**DOI:** 10.1186/s12978-026-02271-7

**Published:** 2026-02-03

**Authors:** Otobo I. Ujah, Onome C. Nnorom, Homsuk E. Swomen

**Affiliations:** 1 Department of Obstetrics & Gynaecology, Federal University of Health Sciences, Otukpo, Nigeria; 2 Department of Community Medicine, Jos University Teaching Hospital, Jos, Nigeria; 3 Ideal Health Consults and Services, Federal Capital Territory, Abuja, Nigeria

## Abstract

**Background:**

On June 24, 2022, the U.S. Supreme Court’s decision in *Dobbs v. Jackson Women’s Health Organization* overturned *Roe v. Wade*, a landmark 1973 U.S. Supreme Court decision that established a constitutional right to abortion, raising concerns about its potential impact on sexual and reproductive health equity. In this exploratory study, we analyzed user-generated content on Twitter (now X) to identify emerging themes and sentiments related to contraception in the aftermath of this landmark ruling.

**Methods:**

In this study, we followed a text mining approach using data obtained from Twitter (Now “X”) to conduct emotion and sentiment analysis and topic modeling. We analyzed 26,755 tweets mentioning “contraception”, “contraceptive” or specific contraceptive methods, collected prospectively between June 24, 2022, the date of the landmark Supreme Court ruling, and July 10, 2022.

**Results:**

The average sentiment of the tweets was predominantly negative. Emotion analysis revealed “trust” (21.4%) as the most frequent sentiment, followed by “anticipation” (17.2%) and “fear” (16.8%). Topic modeling identified 15 prominent themes, including the reexamination of fundamental rights, contraceptive coverage, forced pregnancy, reproductive agency and confidentiality.

**Conclusion:**

The study highlights the role of social media in reflecting societal concerns and uncertainties about contraceptive access with shifts in the legislative landscape of women’s sexual and reproductive health and rights and offers implications for policy and practice in addressing barriers to contraceptive access and use in the post-*Roe* Era.

## Introduction

On June 24, 2022, the Supreme Court of the United States, in *Dobbs v. Jackson Women’s Health Organization*, ended the federal constitutional right to legal abortion, returning the authority to regulate abortion to individual states [1, 2]. The *Dobbs* decision triggered global public debates not only about abortion access but also concerning broader issues of sexual and reproductive health and rights. While the ruling primarily pertains to abortion, it has sparked divided opinions and attitudes regarding its repercussions for the federal right to contraception, consensual sexual intimacy, marriage and reproduction [3]. Nadasen contends that the overturning of Roe has profound ramifications, extending beyond abortion to implicate fundamental issues of privacy, sexual autonomy, and individual rights [4]. This premise aligns with the concurring opinion of Justice Clarence Thomas, who suggested that the Supreme Court should revisit prior rulings related to contraception and marriage equality. However, evidence from prior literature underscores the limitations of judicial rulings, including gaps in court cases that fail to address the broader purposes and effects of regulations on abortion access [5]. This highlights the need for a more comprehensive evaluation of the *Dobbs* ruling’s implications for reproductive healthcare and rights.

Access to contraception remains a fundamental aspect of sexual and reproductive health equity [6]. Contraception plays an important role in enhancing individuals’ reproductive autonomy, yet restrictive abortion laws and policies in the U.S. continue to influence women’s access to and choices about contraceptive methods [7–10]. Previously, such restrictions have included a ban on funding for family planning services that provide abortion services and the exclusion of providers from Medicaid [10]. Studies examining the relationship between state abortion policies and women’s contraceptive use patterns have shown mixed results [8]. Some studies have found a link between the use of highly effective contraceptive methods and the extent of abortion restrictions in a given area. For instance, a study by Jacobs and Stanfors showed that women residing in states with restricted access to abortion were 1.4 times more likely to use highly effective contraceptive methods compared to using no method [11]. Conversely, other studies have found no systematic switch to more effective methods with increasing restrictions. Felkey and Lybecker showed that restricting access to abortion did not increase the use of effective contraceptive methods but instead heightened the likelihood of unwanted pregnancies and, consequently, unsafe abortions [12]. Both studies, however, highlight the considerable influence of the abortion policy context on contraceptive use, emphasizing the need to examine contraception-related issues in light of the recent overturn of *Roe v. Wade* (Hereinafter, *Dobbs*). Nevertheless, the extent to which the *Dobbs* ruling has shaped public discourse on access to contraception and reproductive healthcare remains vastly understudied [13].

There is growing interest among researchers in analyzing user-generated content from social media as a valuable big data source for public health research. Social media platforms provide real-time, easily accessible and searchable content, enabling researchers to track evolving public sentiment and behaviors at scale [14–16]. Increasingly, individuals use platforms such as Twitter, Reddit, WhatsApp and Facebook not only to stay informed about unfolding health events and policies, but also to share their experiences, seek advice from others experiencing similar conditions, obtain emotional support and engage in broader public discourse [17, 18]. Over the past decade, digital platforms have become increasingly sophisticated, allowing public health agencies to deliver tailored, real-time messages to specific populations [19]. Their broad reach, interactive nature and ability to rapidly disseminate information make them ideal tools for health communication, especially during emergencies or public health crises. Additionally, the public actively seeks information on these platforms, making them effective for both messaging and gathering feedback or surveillance data.

Compared to conventional data collection methods, social media offers a cost-effective, efficient alternative that also supports participant recruitment and intervention delivery [20, 21]. Importantly, social media content reflects spontaneous reactions to real-world phenomena and avoids design-related biases associated with hypothetical surveys [22]. For instance, public responses to state-level abortion restrictions in Georgia and Texas have been studied using Twitter data, capturing sentiment in real time [22, 23]. In addition, Pleasants and colleagues analyzed user-generated content on Reddit to examine experiences of challenges to abortion access following the *Dobbs* leak [24]. Thus, social media offers a complementary data source that enhances our understanding of public perceptions on topics like *Roe v. Wade* while more traditional approaches are still underway. However, the analysis of large volumes of unstructured text poses significant challenges, including premature sampling, limited context integration and analytic inconsistency [25]. These limitations can be addressed through advanced computational techniques such as natural language processing (NLP) and computational linguistics [25, 26].

Natural language processing (NLP), a branch of artificial intelligence, analyzes human language to extract meaningful insights. Applications of NLP include sentiment analysis, which detects and evaluates emotions and subjective information in text and topic modeling, which uses algorithms to identify and summarize themes within large datasets [27, 28]. For example, computational text analytic methods have been employed to study population attitudes toward contraception [29], sentiments surrounding abortion laws and restrictions, including the *Dobbs* decision [30, 31], abortion restrictions including the recent Supreme Court ruling in *Dobbs* [20] and reactions to public health crises such as the COVID-19 pandemic [32]. Other applications include analyzing public discourse on breast cancer [33] and social justice movements like “Black Lives Matter” [34]. These studies illustrate the utility of computational methods in generating evidence from social media data to inform sexual and reproductive health policies and future research agendas. By leveraging NLP techniques, researchers can systematically and reproducibly analyze large datasets, uncovering salient themes and sentiments that shape public discourse.

Using NLP techniques, we analyzed user-generated content from Twitter (now X, hereinafter referred to as “Twitter”) to gain insight into population-level discourse on contraception within the context of the U.S. Supreme Court’s decision to overturn *Roe v. Wade*. We selected Twitter as our data source because it is one of the most widely used social media platforms, with over 300 million active users posting more than 500 million tweets daily [14]. Its microblogging format allows users to share concise content including text, images and links which makes it an ideal platform for capturing diverse public expressions [35]. Twitter’s open and searchable nature, combined with its real-time flow of information, enables the rapid detection and analysis of public opinion, sentiment, and discourse as health-related events unfold. Moreover, the platform’s longitudinal tractability and relatively easy data access via public APIs make it particularly well-suited for monitoring trends and engagement at scale over time.

Previous research has demonstrated the utility of Twitter data for reproductive health studies. For example, Merz et al. (2021) analyzed temporal trends in attitudes toward contraceptive methods over more than a decade [29]. McMann et al. (2021) investigated abortion pill marketing and sourcing on Twitter after the Dobbs decision [36]; and Huang et al. (2016) explored how users share contraceptive content on Twitter [37]. Together, these studies highlight Twitter’s value as a tool for capturing dynamic conversations and public reactions, particularly during shifts in legislative and sociopolitical landscapes.

### Theoretical framework

To improve understanding of public discourse on contraceptives within the context of abortion access restriction, this study draws on the psychological reactance theory (PRT). The psychological reactance theory provides an explanation regarding the rationale for and approaches to resist or counter forceful messages at persuasion [38]. Based on this theory, individuals would express motivation to restore specific behavioral liberties (real or apparent) at any time these freedoms are threatened or eliminated [39]. This motivation (reactance) includes negative feelings such as the expression of arguments against freedom restrictions as well as negative cognitions.

Limiting access to the full range of reproductive health services including abortion has been frequently described as a violation of human rights that as well, precludes reproductive justice [40]. Hence, with the Supreme Court’s ruling in *Dobbs*, individuals may perceive or experience threats to their reproductive freedoms not only regarding access to abortion care and services but also contraceptives. Consequently, this results in behavioral and psychological attempts to restore one’s freedom which is complemented by the experience of emotions. Such emotions, beliefs and thoughts are increasingly expressed on social media platforms as a means of communicating and sharing opinions and attitudes about a given social event [41].

The PRT has been widely applied in public health and communication research to explain how individuals respond to perceived threats to autonomy, particularly in areas involving health behaviors, policy restrictions and controversial sociopolitical issues. In the context of social media research, researchers have leveraged PRT to analyze online reactions to public health mandates, such as vaccine policies [42, 43] and alcohol guidelines [44], demonstrating that message framing that triggers perceived threats to autonomy predictably elicits negative sentiment and oppositional discourse on platforms like Twitter. PRT has also been used to analyze public reactions to government policies and other health communication efforts [45, 46].

In the present study, PRT provides a critical conceptual lens for interpreting public discourse on contraception in the post-Roe landscape. By grounding our analysis in PRT, we are able to improve understanding of sentiments and themes regarding the emergence of emotional and discursive patterns following the *Dobbs* decision. Specifically, the theory helps illuminate how expressions of anger, fear, uncertainty or resistance observed in the data may reflect underlying perceptions of threatened reproductive autonomy. Thus, incorporating PRT strengthens the explanatory depth of this study, allowing us to contextualize social media discourse within a well-established theoretical model of human motivation and resistance to freedom-threatening policies.

Building on this theoretical foundation, the present study leverages PRT to examine how the *Dobbs* ruling shaped public debates regarding on access to contraception, highlighting key concerns in the post-*Roe* era to inform agenda-setting and policymaking in sexual and reproductive health. Guided by this framework, we addressed the following research questions:

Research Question 1: What sentiments and emotions were expressed about contraception and contraceptives on social media in response to the reversal of *Roe v. Wade*?

Research Question 2: What themes related to contraception and contraceptives emerge in social media conversations following the reversal of *Roe v. Wade*?

## Methods

**Fig. 1:**
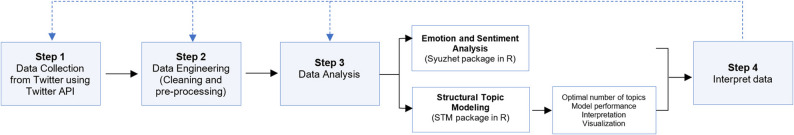
Flowchart illustrating the data collection, cleaning and analysis process

In this study, we employed a systematic approach to conduct textual analysis, which included data collection, data cleaning and preprocessing and data analysis (Fig. 1). All steps were carried out using R software version 4.2.2.

### Data collection

We analyzed data obtained from Twitter (now X), hereto referred to as “Twitter”, one of the most popular social media platforms with over 330 million active monthly users and approximately 500 million tweets produced daily [47, 48]. Data collection spanned from June 24th, 2022 (the date of the Supreme Court ruling) to July 10th, 2022, using Twitter’s official API and the “twitteR” library in R. This period was chosen as the topic of *Roe v. Wade* became a trending subject on Twitter, reflecting public reactions to the *Dobbs v. Jackson Women’s Health Organization decision*. We chose to end data collection on July 10, 2022, because tweet volume had significantly decreased and plateaued by that point, indicating the end of the immediate surge in public discourse following the Dobbs decision. Extending data collection beyond this period would have captured substantially fewer new insights and introduced diminishing returns for qualitative and quantitative analysis.

Tweets were initially retrieved using keywords, phrases and hashtags related to Roe v. Wade, such as “Roe”, “Roe v. Wade”, “Roe vs Wade”, “#Roe”, “#roevwade”, “roevswade” and “roeoverturned”. As our focus was on conversations surrounding the *Dobbs* decision and contraception, we extracted a subset of tweets discussing contraception/contraceptives using keywords associated with birth control, including “contraceptive”, “contraception”,” iud”, “iucd”, “emergency contraception”, “emergency contraceptive”, “birth control”, “birth control pills”, “plan b”, “planb”, “mirena”, “implant”, “injectables”, “condoms”, “injectable”, “morning after pill”, “copper iud”, “tied tubes”, “LARC”, “vasectomy” and/or “tubal ligation”. The choice of keywords was guided by a combination of author consensus and an examination of tweets and internet content related to the *Dobbs* decision, as has been done in previous studies [49–51]. We restricted the study to English-language tweets, retaining only original tweets while excluding retweets from the analysis. However, quoted retweets were not treated as simple retweets. Instead, they were included in the analysis as original tweets since they contain additional user-generated content and reflect unique user engagement. This distinction was made to better capture how users interacted with contraception-related content through both amplification and commentary.

### Data preprocessing and cleaning

Prior to data analysis, preprocessing and cleaning techniques were applied to remove irrelevant elements and prepare the text corpus for analysis. This process involved eliminating special characters, usernames, hyperlinks, punctuation and stop words. In NLP, stop words are commonly occurring words that appear frequently in most documents but contribute minimal semantic value, such as “*and*”, “*the*”, “*is*”, “*in*” and “*of*” [52]. All tweets were converted to lowercase and tweets with four or less words were excluded as they do not provide useful semantics [53]. To ensure the anonymity of Twitter users, Twitter handles were replaced with “@username” when presenting phrases and quotes. Additionally, to preserve the meaning of key phrases that could be lost during tokenization (the process of breaking tweets into individual units), these phrases were converted into single-word representations that retained their original meaning (“supreme court” to “scotus”, “pro life” to “prolife”, “pro choice” to “prochoice”; see Table 1) [50]. Also, we used the grepl() function in R to detect and remove tweets that included terms commonly associated with petition campaigns. For example, tweets containing keywords such as “insurrectionists” and “abortion pill” were filtered out.

**Table 1:** Examples of ‘raw tweets and their processed version prior to topic extraction

Raw tweet	Processed tweet
REMEMBER! THIS IS A SLIPPERY SLOPE! #RoeVsWade WONT BE THE LAST! You know what comes next, right? They’ll repeal same-sex marriage, interracial marriage, legality of contraceptives and birth control, equal rights, voting rights. it won’t end with this.	remember slippery slope repeal gay marriage interracial marriage legality contraceptives birth control equal rights voting rights wo
Deeply saddened by this. It’s hard to accept that actual villians have a minority rule. Women have less rights today than they did 49 years ago when Roe v Wade was decided. Next is birth control and contraceptives https://t.co/h62HsNkT9q	deeply saddened hard accept actual villians minority rule women rights ago decided birth control contraceptive
I am scheduled for a hysterectomy next Friday because of prolapsed uterus and I am about to throw away 8 unopened packs of birth control. It just feels wrong in light of the decisions the #SCOTUS is making #RoeVWade #RoeVsWade #women #birthcontrol	scheduled hysterectomy friday prolapsed uterus throw unopened packs birth control feels wrong light decisions
This is a very touchy subject. I personally don’t agree with abortion, but I believe the solution lies within sex education, access to Plan B and contraceptives, etc. I agree with the original Roe decision in that it should be legal up to a point, THEN states can regulate it(2/?)	touchy subject personally agree abortion solution lies sex education access planb contraceptives agree original decision legal regulate
People are buying into a fear campaign. No one is coming for same-sex marriage, contraceptives or private sexual acts. Roe was unique because it involved the lives of the unborn. https://t.co/lWqZw17Qxd	people buying fear campaign coming gay marriage contraceptives private sexual acts unique involved lives unborn
Well done to the US SupremeCourt ruling. I support it but it must be balanced against medical or circumstantial reasoning. I do not support abortion as a means of birth control. Now just that 2nd amendment & Gun control needs reform too. 👍 #Roe	scotus ruling support balanced medical circumstantial reasoning support abortion means birth control amendment gun control reform

### Data analysis: exploratory data analysis, sentiment analysis and topic modeling

We summarized the quantitative characteristics of tweets using descriptive statistics, including mean and standard deviation (SD) for continuous variables, as well as frequencies and percentages (%) for categorical variables. A word cloud was generated to visualize the 100 most frequently occurring words, with font size representing their frequency within the corpus of tweets. Sentiment and emotion analysis, along with topic modeling, were then performed to classify sentiments, identify emotions and uncover key themes in the dataset.

#### Sentiment and emotion analysis

We performed sentiment and emotion analysis of the library of tweets. Sentiment analysis, a NLP technique, was employed to extract subjective information from unstructured text and detect the tone’s polarity (positive, neutral or negative) using computational algorithms [54, 55]. While sentiment analysis approaches can be categorized as lexicon-based or machine-learning methods [56], in this study, we used the lexicon-based approach. Specifically, the “syuzhet” library in R was applied to identify prevalent sentiments and emotions within the dataset. Emotion analysis was performed using the NRC Word-Emotion Association Lexicon dictionary, which contains 14,182 words and classifies emotions based on Plutchik’s wheel of emotions into eight categories: anger, anticipation, disgust, fear, joy, sadness, surprise and trust [57–59]. This approach enabled a deeper understanding of the emotional expressions embedded in the tweets.

#### Topic modeling

Topic modeling is an unsupervised machine learning technique used to uncover the underlying thematic structure within a collection of documents by employing hierarchical probabilistic models [60]. To identify emerging themes in Twitter conversations about contraception in relation to the overturning of *Roe v. Wade*, structural topic modeling (STM) was employed using the stm package in R. Unlike conventional approaches such as Latent Dirichlet Allocation (LDA) and Latent Semantic Analysis (LSA), STM provides insights into latent topic structures by analyzing word co-occurrence patterns using a bag-of-words representation while incorporating document-level metadata [61–63]. Additionally, STM facilitates the evaluation of covariate effects on topics [59]. In this study, each tweet was treated as a document and sentiment polarity served as the covariate. The approach to thematic analysis applied in this study involved the process of (i) selecting the optimal number of topics (ii) building the topic model (iii) visualization, labelling and explanation of the topics and (iv) topic clustering.

The optimal number of topics (k) was determined by incrementally building topic models with k values ranging from 5 to 50 in increments of five. The models were evaluated using four common metrics – exclusivity, semantic coherence, residuals and lower-bound likelihood. Exclusivity in STM measures how uniquely the top words in a topic belong to that topic, rather than being shared across multiple topics [64]. High exclusivity indicates greater topic distinctiveness and interpretability. In contrast, semantic coherence reflects the degree to which the most probable words within a topic co-occur frequently in documents and make intuitive sense as a group [65]. High coherence suggests a topic is internally consistent and meaningful. In STM, topics that are both highly cohesive (words co-occur) and highly exclusive (words are unique to the topic) tend to be the most semantically useful and interpretable [66].

The final selection of k was guided by these metrics and the examination of an exclusivity-semantic coherence plot. Each topic was interpreted based on metrics from the STM output, including highest probability, FREX (a combination of frequency and exclusivity), score and lift. The authors independently reviewed the word association indices for each topic and defined the topics accordingly. Final topic labels were determined by reconciling similarities in the definitions provided by both authors. A correlation network was then used to visualize the topics and explore their interrelationships, providing further insights into the thematic structure of the dataset.

## Results

### Descriptive results

Between June 24, 2022 and July 10, 2022, our keywords identified a total of 3,161,353 Roe-related tweets. From this dataset, 32,960 unique tweets containing words or phrases related to contraceptives were extracted. Of these, 13.1% (*n* = 4,231) contained petitions, which were excluded, using a keyword-based filtering approach, to minimize potential distortion of public discourse and reduce bias in the analysis. This exclusion resulted in a sample of 26,897 tweets. Further filtering of tweets with fewer than four words produced a final analytic sample of 26,755 tweets from 22,847 unique Twitter users.

**Fig. 2:**
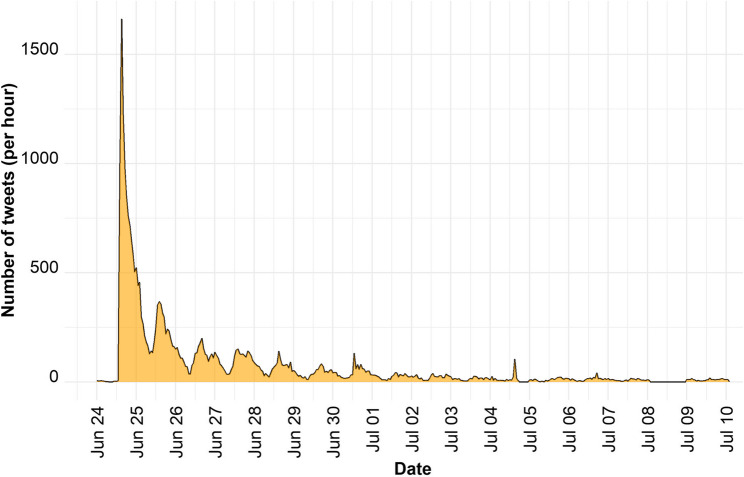
Number of contraception-related tweets in conversations regarding the Dobbs decision during the study period

Approximately 90% of the unique user accounts posted only one tweet. The number of followers per user account ranged from 0 to 58,769,303, with a median of 333 followers. The tweets analyzed in this study were retweeted an average of 3.4 times (SD = 75.5) and the mean number of favorites by Twitter users was 4.0 (SD = 357.6). The temporal distribution of tweets related to contraception/contraceptives following the reversal of *Roe v. Wade* showed distinct patterns of public discourse over the study period (Fig. 2). Tweet activity peaked sharply on June 24, 2022, coinciding with the day of the ruling, with the highest hourly volume observed during this initial period. In the days immediately following June 24, tweet volume declined rapidly but remained higher than baseline levels. By June 27, the rate of tweets stabilized, maintaining a consistent but lower level of activity throughout the study period. Despite the stabilization, periodic increases in tweet activity were noted on June 30, July 4 and July 5, which may correspond to secondary discussions or events related to the *Dobbs* decision. The average number of tweets per user account during the study period was 1.17 (SD = 1.02).

### Word frequency

**Fig. 3:**
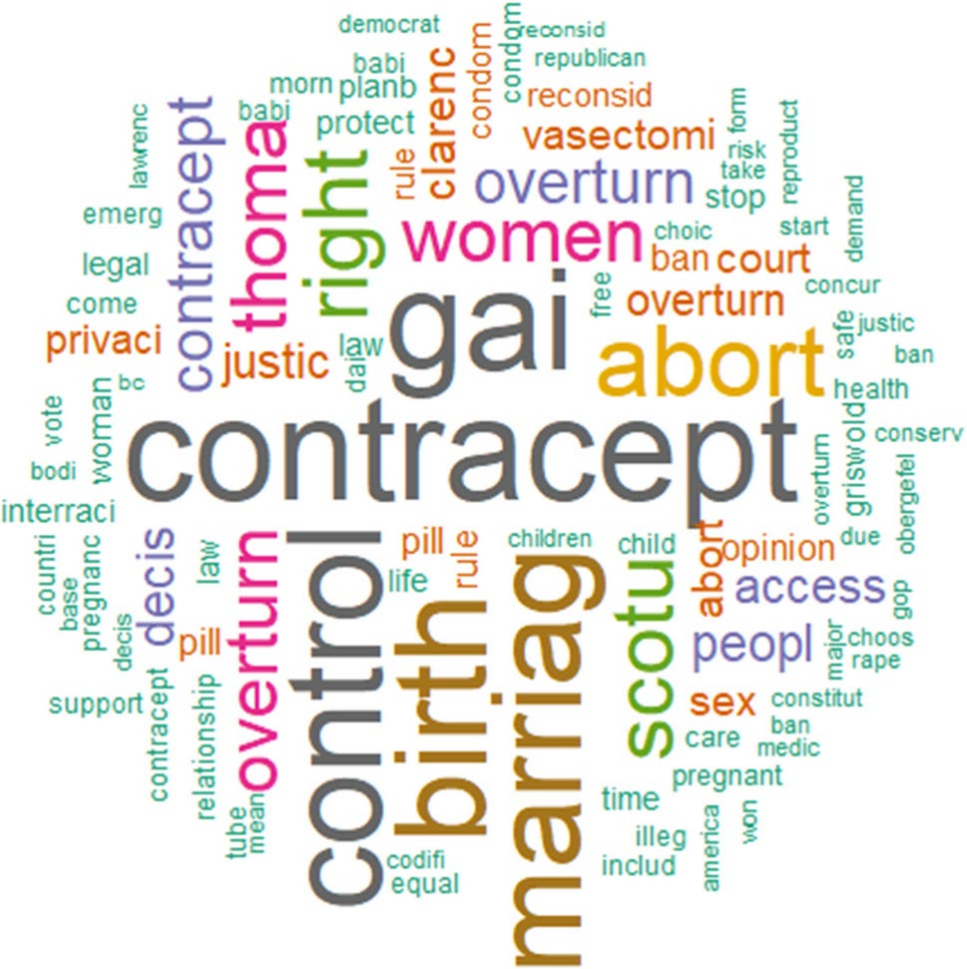
Word cloud visualizing the most frequent terms in the tweet corpus discussing contraception post-*Dobbs*. The size of each word corresponds to its frequency within the dataset of 282,062 unique words, with “contraception” being the most prominent term

Word frequency analysis of the corpus, comprising 282,062 unique words, revealed key themes in the discourse. The most prominent terms are visualized in the word cloud (Fig. 3), which highlights “contraception”, “birth control”, “marriage”, “abortion”, “rights” and “SCOTUS” as central to the discussion.

### Sentiment polarity and emotions

**Fig. 4:**
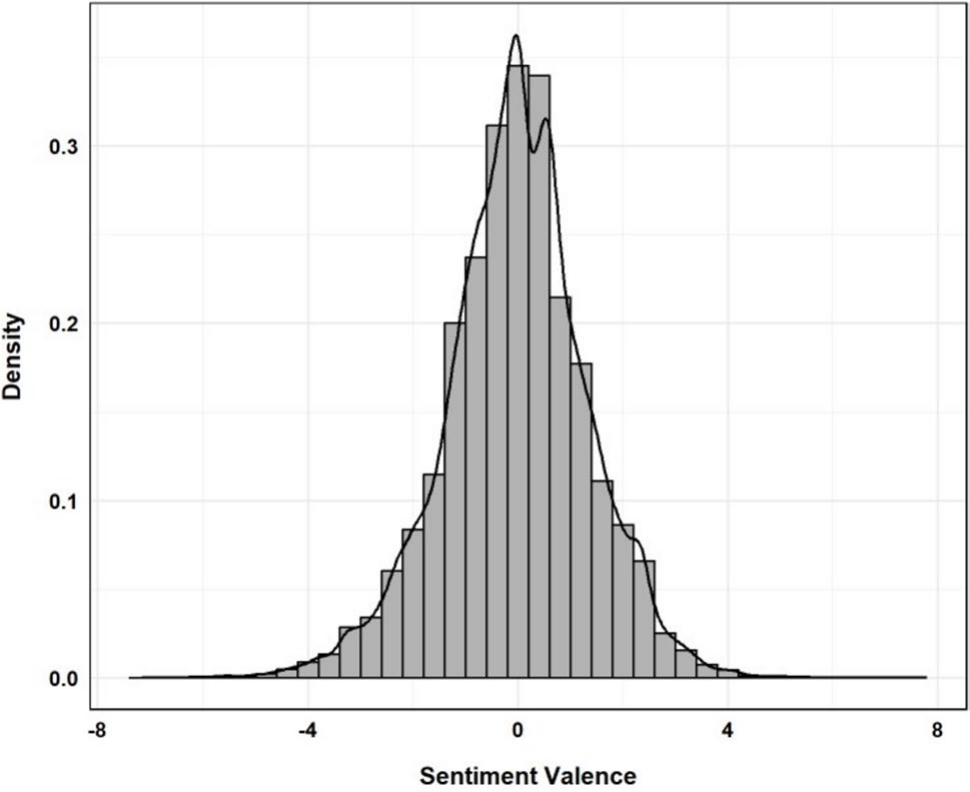
Histogram of sentiment values within the Twitter corpus. Positive values on the x-axis represent positive sentiment, while negative values indicate negative sentiment

Figure 4 illustrates the distribution of overall sentiment scores (valence) in the corpus of tweets. Sentiment values less than zero indicate negative sentiments, values greater than zero indicate positive sentiments and values equal to zero represent neutral sentiments. Among these three categories, negative sentiments accounted for the largest proportion (48.2%), narrowly surpassing positive sentiments (47.4%). Neutral sentiments comprised only 4.2% of the total.

Additionally, of the eight emotions analyzed, trust was the most frequently expressed emotion across the corpus, appearing in 21.5% of the tweets. This sentiment was often conveyed through language expressing certainty, belief, and reliance on a particular outcome or state of affairs. For example, the tweet “*The Supreme Court decision on Roe vs. Wade will certainly get women’s attention that need to be on birth control. The use of birth control will be employed more vigorously and often if abortions are illegal. I believe that to be a fact.”* employs markers of conviction to express trust in a predicted causal relationship. Similarly, the statement, “*If you are privileged enough to have foolproof contraception*,* do not take it for granted. Far too many people can not afford IUD’s or are steadily losing access. Real lives are on the chopping block now. If you have access to an IUD you are one of the lucky ones. RoeVsWade*” frames reliable contraception as a trusted safeguard that imply a confident reliance on a given contraceptive method.

Anticipation was the next most prevalent emotion, accounting for 17.2% of the emotions expressed. Tweets in this category were characterized by language focused on looking forward to, predicting, or preparing for future outcomes. Examples include:

*@username @username Not exactly the same: like everyone*,* I saw the writing on the wall for Roe when Trump was elected. My confident prediction for birth control is that arguably abortifacient methods will be targeted in some states*,* while condoms*,* etc. will never be seriously threatened in any state*.

and

*Some hopeful news in the decision overturning Roe V Wade is the majority opinion stated that it should not be taken as an indication of the court reconsidering other unenumerated rights like same-sex marriage or sexual activity*,* contraception or birth control.*

These signal anticipation through verbs of forecasting while also analyzing the majority opinion for clues about future judicial actions, engaging in an act of expectation and preparedness.

Fear was the third most common emotion, expressed in 16.8% of tweets. This was identified through direct mentions of anxiety, threat and negative apprehension. Examples include:

*I already have a fear of getting pregnant and having kids and the overturn of roe v. Wade is giving me anxiety… because I wanted to stop taking my birth control now I’m definitely not. Even if California remains safe*.

and

*Not me getting my birth control removed last week and roe v wade being overturned today. that’s a bad omen I think*.

Joy was the next most prevalent emotion, representing 18.4% of tweets. In this context, joy is often manifested as relief or gratitude in the face of a broader negative event, rather than unalloyed happiness. Examples include:

“Just happy that me and mine have extended forms of birth control and we live in CA. As frustrated and scared as I am, I can’t imagine the way those without extended birth control and living else where are feeling rn, with literally no choice. #RoeVsWade “.

and

My MIL and I were discussing Roe V Wade being overturned and she was talking about how she was glad it was because she’s “with God” —she told me that if a kid were to get raped, took a pill, and it didn’t work-that baby is a blessing from God…I am shocked to say the least.—.

and

I am blessed enough that my husband looked at me and said “Fuck this shit, make me an appointment for a vasectomy.” The second the #RoeVsWade news came. Appointment made within an hour.

**Fig. 5:**
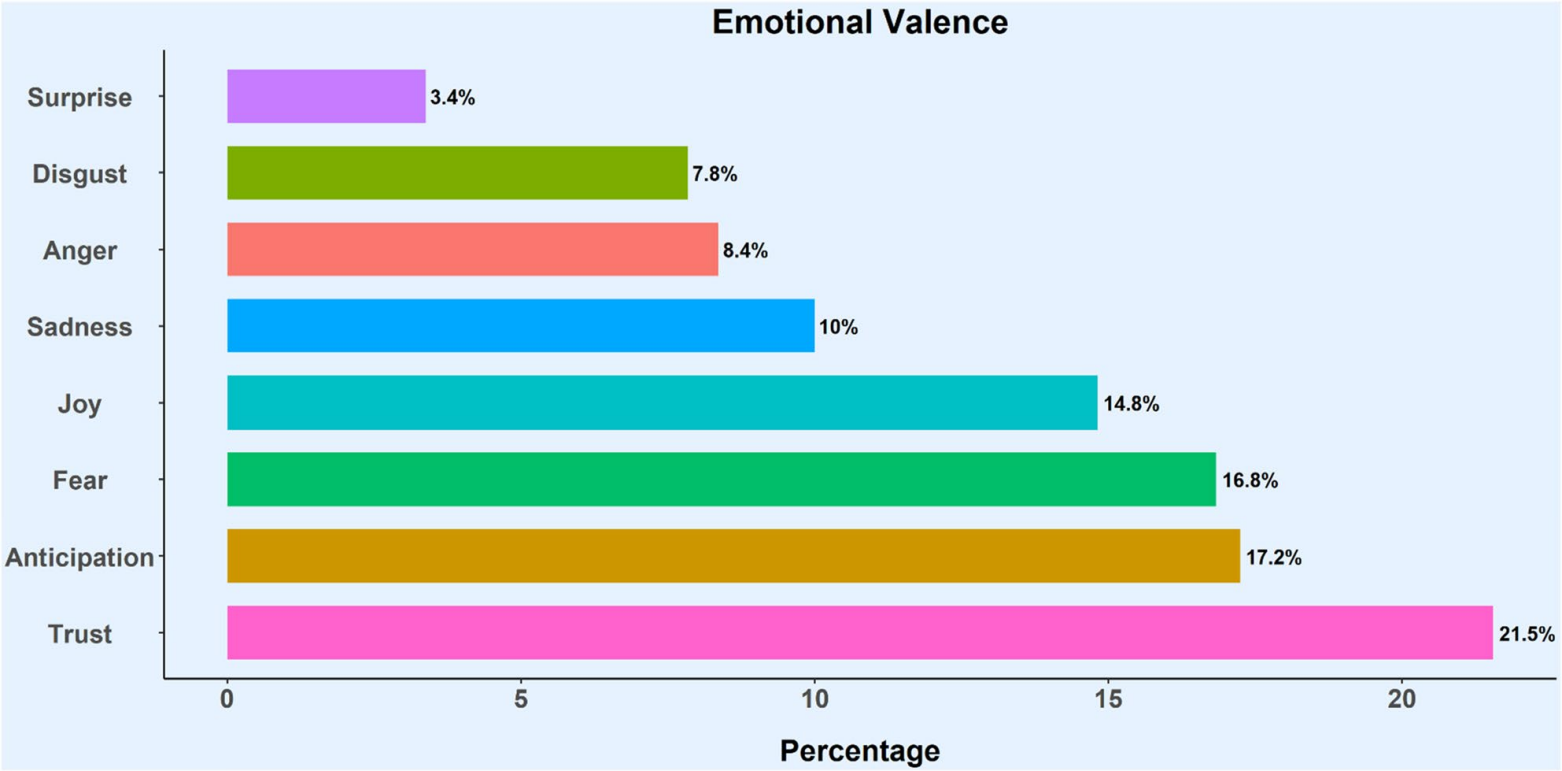
Analysis of emotions in contraception-related tweets using the Syuzhet package in R, based on the NRC-word lexicon

The less common emotions observed were related to disgust (7.8%), often expressed through language of revulsion or moral condemnation; anger (8.4%), characterized by frustration and outrage; and surprise (3.4%), indicating shock or unpreparedness for the event. Figure 5 illustrates the eight emotions associated with contraceptive discourse and their relative frequencies in the corpus of *Roe*-related tweets.

### Topic modeling

**Fig. 6:**
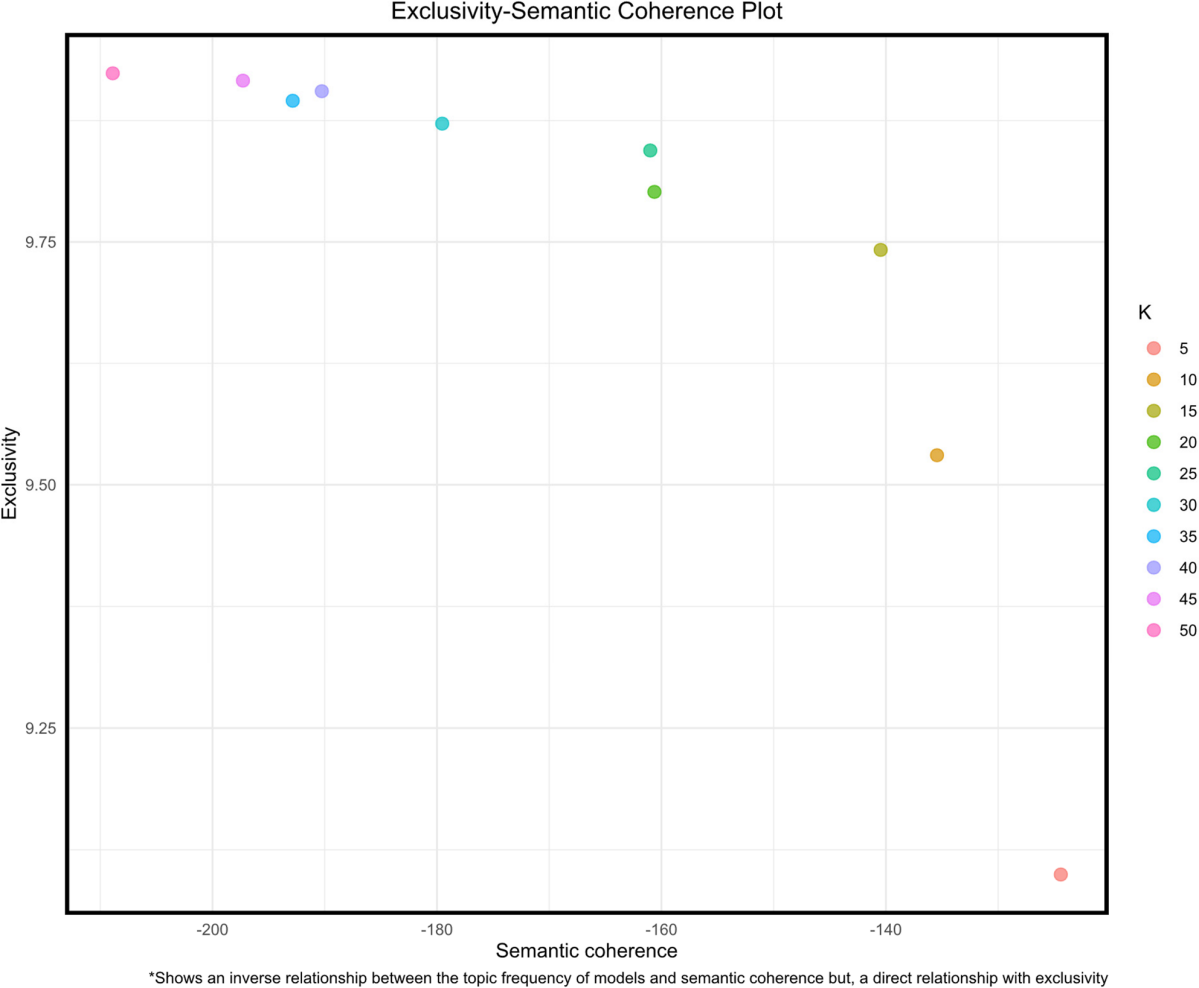
Exclusivity-Semantic Coherence Plot showing the relationship between semantic coherence and topic exclusivity for different topic models. Each point represents a model with a specific number of topics (K), as indicated by the color gradient. Higher exclusivity scores indicate more distinct topics, while higher semantic coherence scores indicate more interpretable topics. An inverse relationship is observed between semantic coherence and the number of topics, with models featuring fewer topics generally demonstrating greater semantic coherence

**Fig. 7:**
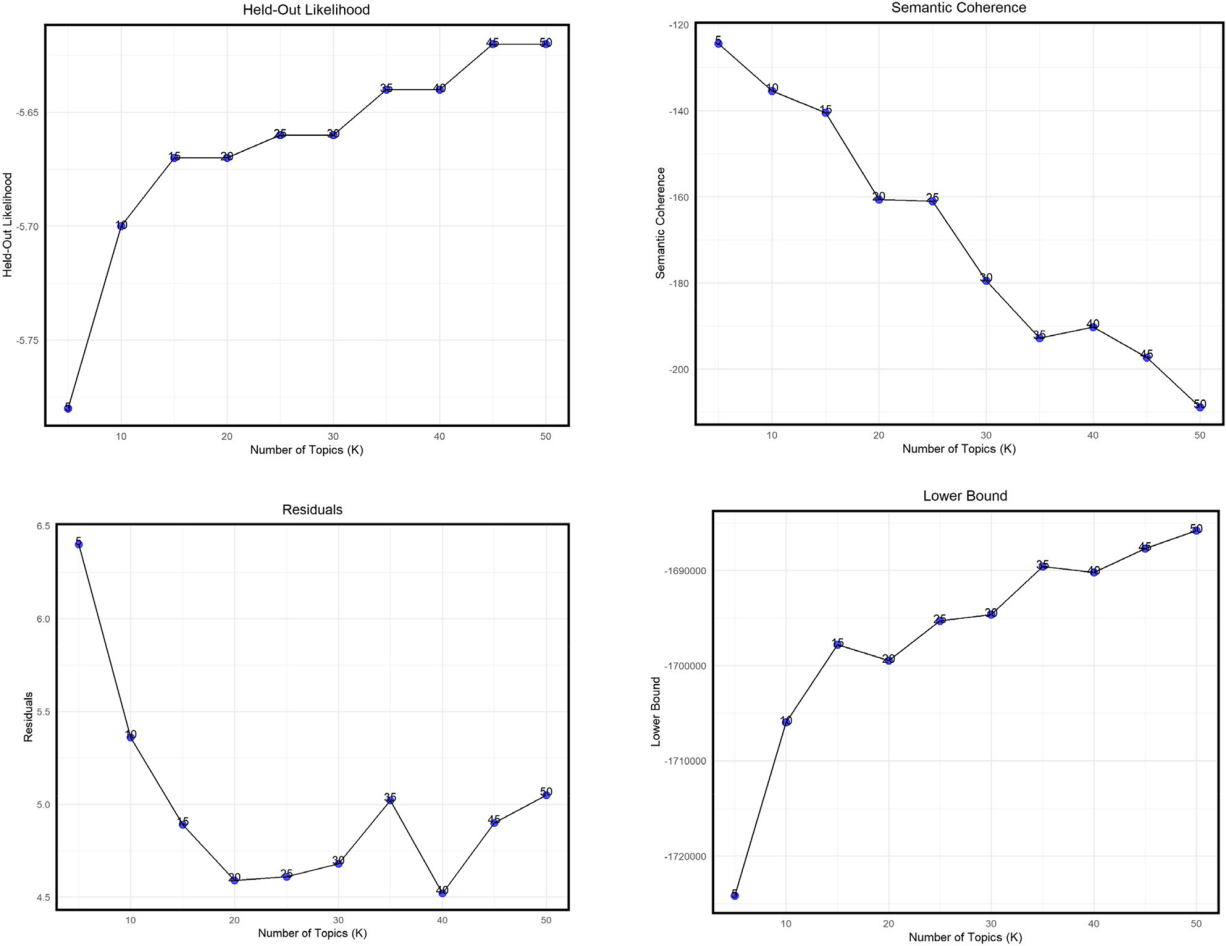
Diagnostic metrics for 5–50 topic models

**Fig. 8:**
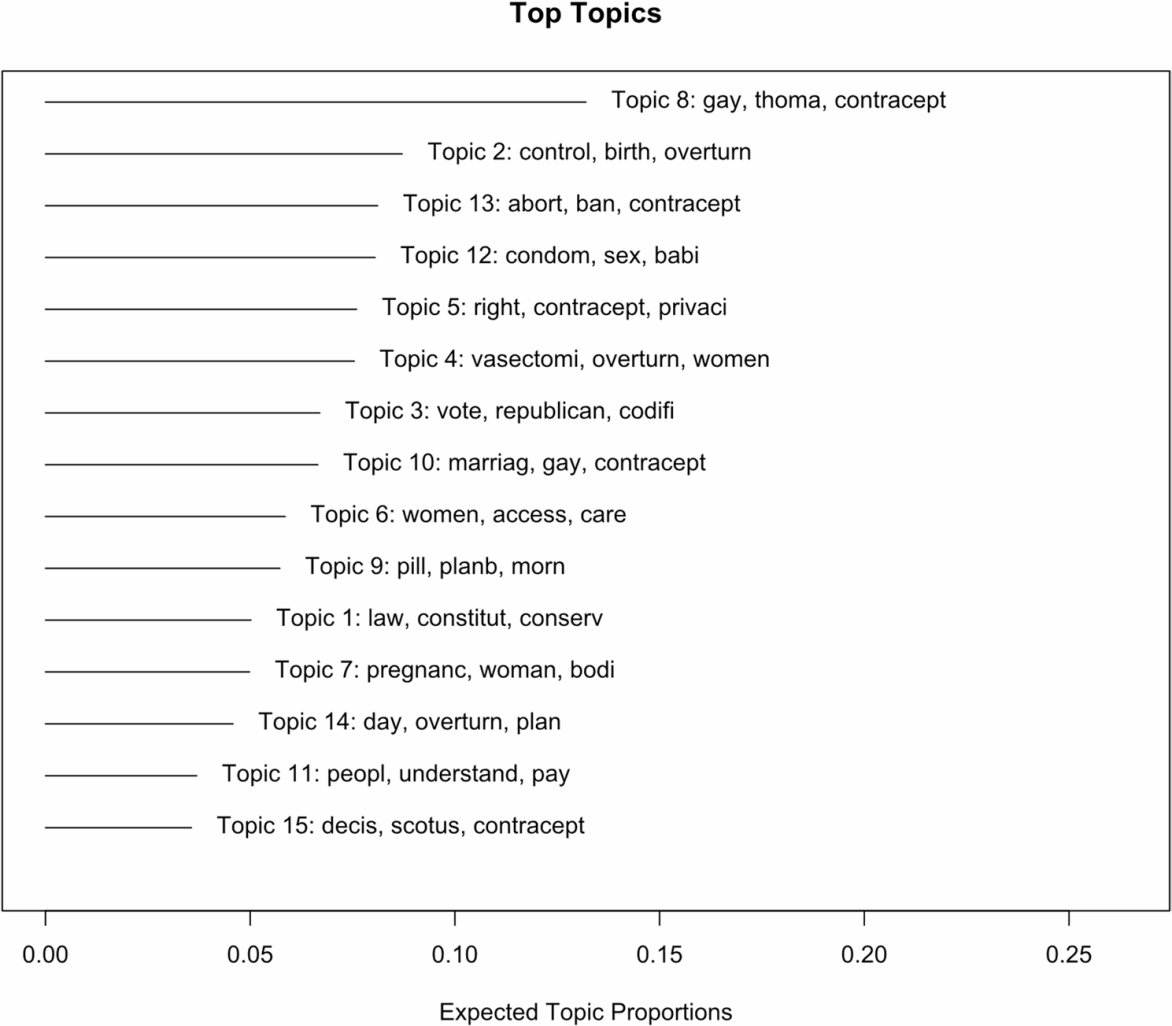
Topic proportions of contraceptive discourse in the corpus of *Roe*-related tweets

Figure 6 displays exclusivity and semantic coherence values from an STM analysis of the tweet corpus, exploring models with 5–50 topics. Based on diagnostic metrics (exclusivity, semantic coherence, held-out likelihood and residuals) shown in Fig. 7, a 15-topic model was deemed the best fit. Figure 8 displays the expected topic proportions, which indicate the relative share of the conversation devoted to each theme. Higher values indicate that a larger proportion of tweets in the corpus were associated with that topic. For example, Topic 8 (gay rights, Justice Thomas, contraception) and Topic 2 (birth control, overturn) accounted for the largest shares of discourse, showing that concerns about broader civil rights and contraceptive access were central to post-*Dobbs* discussions. By contrast, topics such as discounts on emergency contraception (Topic 11) and scaremongering claims (Topic 15) were discussed far less frequently, suggesting these were more niche conversations. Overall, the distribution of topic proportions highlights that the public debate on social media was dominated by rights-based concerns and access issues, while practical or economic aspects of contraception were less prominent. Table 2 provides detailed topic labels. Key themes emerged from discussions around *Dobbs* decision, particularly those mentioning contraception or specific methods. Approximately one-third of the discourse centered on reexamination of fundamental rights (Topic 8), contraceptive coverage (Topic 2), forced pregnancy (Topic 13), reproductive agency (Topic 12) and confidentiality (Topic 5). Lesser-discussed topics included discounts on emergency contraceptives (Topic 11) and scaremongering claims (Topic 15).

#### Reexamination of fundamental rights (topic 8)

This topic encompasses discussions focused on Justice Clarence Thomas’s concurring opinion and its implications for reconsidering established privacy-based rights. Justice Clarence Thomas’s concurring opinion called for reconsideration of past rulings codifying rights to contraceptive access and same-sex relationships.

##### Example 1

“clarence thomas wants to come for same sex marriage & contraception bc those cases are based on the same legal framework as roe v wade but so is the loving case, which legalized interracial marriage, wonder why he didn’t mention that one???? 🤔”.

##### Example 2

“After the Supreme Court announced its decision to reverse Roe v. Wade, Justice Clarence Thomas urged his colleagues to reevaluate other landmark cases protecting contraceptive access and same-sex marriages on the same bases. [link]”.

#### Contraceptive coverage (topic 2)

This topic is defined by concerns about insurance coverage for contraceptives becoming restricted or eliminated, creating financial and access barriers. Discussions highlighted insurance limitations affecting contraceptive access. There were also claims that some contraceptive methods may become unavailable under health insurance.

##### Example 1

“once news broke about roe vs wade i called the doctor about my birth control because this isn’t just about abortions this is also going to take away my right for birth control & they said they’re not refilling birth control at this time until the new insurance policies come in 🙃”.

##### Example 2

“Morning Beautiful People just a FYI. Please spread the word because of the over turn of roe v wade. As of Aug 2022 certain contraceptives will nolonger be free under health insurance. You will be responsible for cost sharing. Not sure who needs to hear this but please past on.”

#### Forced pregnancy (topic 13)

This topic captures the specific concern that contraceptive access is insufficient in cases of sexual violence, where pregnancy is not a choice. Within this theme, uncertainty arose about contraceptive access in cases of forced pregnancy (rape or incest).

##### Example 1

“Most STATEs one can get an abortion on grounds of rape or incest. Morning after pills are commonly used now! Tempest in a teapot over the Supreme Court correctly admitting their Roe legislation was wrong! Just like the Supreme Court did in Board of Education overruling Plessy.”

##### Example 2

“For people who inevitably say just use birth control, it’s not 100% effective. It doesn’t help in the case of rape or incest. And the laws dictating the rights to use contraceptives are based on Roe. IUDs are already set to become illegal in some states. [link]”.

#### Reproductive agency (topic 12)

This topic centers on debates about personal responsibility, framing the use of contraception and abstinence as individual moral choices to prevent pregnancy. This topic featured varying opinions about abstinence and the use of contraceptives to prevent unintended pregnancy as well as personal responsibility of individuals to avoid pregnancy.

##### Example 1

“@username @username @username So she has to make responsible decisions about sex and contraception now, oh the horrors! Speaking of horrors, how about the 63 M babies that were killed since Roe, infinitely more compelling than this woman’s horror at not being able to be irresponsible”.

##### Example 2

“@username I see nowhere in the Constitution a RIGHT to end the life of an inconvenient child. If that woman is smart enough to wear a damn mask for 2 years, she should be smart enough to give her boyfriend a friggin CONDOM! or take a pill! to PREVENT the pregnancy #RoeVWade”.

#### Confidentiality (topic 5)

This topic focuses on the legal argument that *Roe* was built on a right to privacy, and its overturn jeopardizes the foundation for other rights like contraception and marriage equality. This topic highlights the shared legal foundation of *Roe v. Wade* with other federal rights, such as the right to privacy, access to contraception and marriage equality. Concerns were expressed that the Supreme Court’s decision to overturn Roe could undermine these foundational rights.

##### Example 1

“The precedent that Roe is built on top of is the same one that protects the use of contraceptives, the right to marry, and the right to not be sterilized by the state. To refute “the foundation” that Roe is based on equally casts those rights aside, which is reprehensible”.

##### Example 2

“16/…help is available. Where contraception and family planning - which might have prevented the unwanted pregnancy in the first place - are also not funded by the state. Instead, the right wing’s gutting of Roe’s right to privacy is also a gutting of the right to contraception.”.

#### Permanent contraceptive methods (topic 4)

This topic reflects a surge in interest and anxiety around permanent solutions like vasectomies and tubal ligations as a direct response to the *Dobbs* decision. This topic reflects concerns about how the overturning of *Roe v. Wade* may lead individuals to pursue permanent contraception like vasectomy or tubal ligation.

##### Example 1

“There’s been a majority uptick in vasectomy requests to urologist in the USA, especially for men around the age of 30. Good but I have a question for these American men. Why didn’t you have vasectomy before Roe vs Wade was overturned?”.

##### Example 2

“Well I guess with Roevwade overturned the next step is getting your tubes tied then or is the conservatives going to also take that away from women also and really be strick and we live in the wild west 1800s again FUCK THE USA I SAY”.

#### Firearm regulation (topic 3)

This topic is defined by a comparative argument that gun rights are being prioritized over and are expanding faster than reproductive rights. Discussions centered on prioritizing gun control over restrictions on sexual and reproductive rights.

##### Example 1

“I’m so furious I could cry! Racism, no gun control, now no women’s rights? And they want to visit same sex marriage and contraception. This is not just a fight for women!! Everyone needs to fight. This country & their fake ass idea of Christianity. #RoeVsWade”.

##### Example 2

“We live in a country where guns have more rights than a human being. Overturning means jeopardizing protecting contraception, same sex relationships/marriages. This is a way to control, stripping us of our bodily autonomy. This is not normal. It should never be normal #RoeVsWade.”

#### Marriage equality (topic 10)

This topic captures the fear that the legal reasoning behind overturning *Roe* creates a precedent to revoke rights to same-sex and interracial marriage. Many voiced concerns that the overturn of *Roe* could set a precedent to challenge same-sex and interracial marriage rights.

##### Example 1

“This is about losing our right to gay…interracial marriage…to have access to contraception, none of that is enumerated in the Constitution and the Supreme Court has just shown us that they don’t care and they can take that from us.” #RoeVsWade [link]”.

##### Example 2

“@username @username @ sername @username @username @username He also just opened the door for bigots to fight to over turn gay rights, same sex marriage, contraception access and, even though he didn’t say it aloud, interracial marriage (as all those cases from Loving to Griswold use the rights established by the 14th, just like Roe did).”

#### Women’s health access (topic 6)

This topic broadly discusses the landscape of reproductive healthcare access, including contraception and abortion, in a post-*Roe* world. This topic included concerns about access to contraception and reproductive health care following the *Dobbs* decision.

##### Example 1

“Like all euphemisms and mental gymnastics the family planning & care title is only that. If you need reproductive health you’ll get it in a post Roe v wade world. Just be responsible use contraceptives so you don’t create a little one. Religious or not abortion is abhorrent”.

##### Example 2

“@username Probably would. I think 2.) things were squished in the #RoeVsWade. (1) Debauchery, or irresponsible living, and (2) giving power back to the states. I picked SD to live, & its heavy GOP, but I am indie. I vote for who I like, policy wise. Abortion here, is birth control. 99% “.

#### Emergency contraceptive (EC) surge (topic 9)

This topic documents the immediate consumer response to the decision: a spike in demand for emergency contraceptives leading to shortages and purchasing limits. This topic captures the surge in demand for emergency contraceptives post-*Dobbs*, leading to supply shortages and purchasing limits.

##### Example 1

“@username Let’s try using a few of the many forms of birth control that wasn’t easily available when *Roe* vs Wade was signed. I get that this is emotional but please think about what is available now to prevent pregnancies, including plan b and the morning after pill”.

##### Example 2

“Demands for emergency contraceptives have surged following the Supreme Court’s decision to overturn Roe vs. Wade. It’s led a few pharmacies to start limiting the number of emergency contraceptive pills customers can purchase. @usernmae joins @username to discuss. https://t.co/tqe97klOLN”.

#### Political influence (topic 1)

This topic is characterized by critiques of the Supreme Court’s motives, framing the decision as an act of political and religious ideology rather than sound legal judgment. This theme critiques the Supreme Court’s decision as politically and religiously motivated rather than grounded in legal reasoning.

##### Example 1

“The Supreme Court is nothing more than a bunch of conservatives using the constitution as a political tool. Not only have they overturned Roe v. Wade, but they limited enforcement of Miranda Rights, authorized funding for religion, and are now after contraception and LGBT rights.”

##### Example 2

“ What is most shocking to me is suggesting that birth control be outlawed because it is not written in the constitution. These conservative justices are morons. #RoeVsWade #contraception”.

#### Bodily autonomy (topic 7)

This topic is defined by the argument that individuals have an inherent right to make decisions about their own bodies, encompassing both contraception and abortion. Users discussed the fundamental right of individuals to make choices about their bodies, including contraception and pregnancy decisions.

##### Example 1

“Women need to stop crying about being asked to take accountability for their actions. It’s a simple fix. Your body your choice so stop choosing to sleep around with men that don’t use condoms if you don’t want to get pregnant. This is a woman problem not a man problem. #RoeVsWade”.

##### Example 2

“People, freedom = choice. I’m lucky enough I’ve not been raped and I got pregnant (on the pill + condors) at 29 when I was stable in my career. Same time, I would NEVER remove a woman’s #righttochoose #yourbodyyourchoice #RoeVsWade”.

#### Physician consultations (topic 14)

This topic specifically highlights the reported increase in individuals consulting doctors about permanent contraception procedures post-*Roe*. Under this topic, there were reports of an increased demand for permanent contraception methods for both men and women following the overturning of *Roe v. Wade.*

##### Example 1

“@username I am a republican but I do not agree with roe vs wade being overturned. I had an abortion in 2009 and to this day I did the right thing. I use the implant but it might be time to get my tubes tied.”

##### Example 2

“In light of overturning of #RoeVsWade legislation urologists see spike in requests as people fear states could next move to restrict contraception.Never expected this as a result of it’s overturning. Just another consequence of the repressive legislation. https://t.co/ziiRiqAyer.”

#### Discounts on emergency contraceptives (topic 11)

This topic focuses on the commercial response to the increased demand, specifically online pharmacies offering promotions or discounts on emergency contraceptives. There were claims that, following the reversal of *Roe v. Wade*, online pharmacies began offering discounts on emergency contraceptives such as Plan B.

##### Example 1

“Mark Cuban’s online pharmacy is offering steep discounts on birth control and Plan B–like drugs as people stock up after Roe is overruled [link].

##### Example 2

“Plan B pills literally getting price gouged online as we speak, or selling out completely. To say people are freaking the fuck out is putting it lightly. #RoeVsWade #RoeVWade #SupremeCourt [link]”.

#### Claims of scaremongering (topic 15)

This topic represents a counter-narrative that dismisses concerns about contraception and other rights being threatened as exaggerated or politically motivated “fear campaigns.” Some expressed skepticism, claiming that fears surrounding the overturning of *Roe v. Wade* extending to contraception access were exaggerated or unfounded.

##### Example 1

“People are buying into a fear campaign. No one is coming for same-sex marriage, contraceptives or private sexual acts. Roe was unique because it involved the lives of the unborn. [link]”.

##### Example 2

“In the face of uncertainties and fear post #RoeVWade, people are stockpiling emergency contraceptive pills. Here’s what to know about emergency contraceptive pills, how they’re different from abortion pills, and if they’re still legal. [link]”.

**Table 2:** 15-Topic structure characterizing contraceptive discourse in *Roe*-related tweets

Topic #	Topic labels	Top 10 words	Proportion (%)
Topic 8	Reexamination of fundamental rights	gay, thoma, contracept, overturn, justic, marriag, scotus, rule, clarenc, court	13. 2
Topic 2	Contraceptive coverage	control, birth, overturn, women, effect, option, method, live, free, cover	8.7
Topic 13	Forced pregnancy	abort, ban, contracept, legal, illeg, rape, form, medic, mean, overturn	8.1
Topic 12	Reproductive agency	condom, sex, babi, pregnant, kill, stop, respons, wear, start, murder	8.0
Topic 5	Confidentiality	right, contracept, privaci, griswold, equal, preced, includ, gay, marri, obergefel	7.6
Topic 4	Permanent contraception	vasectomi, overturn, women, fuck, forc, revers, tube, tie, children, kid	7.5
Topic 3	Firearm regulation	vote, republican, codifi, right, america, democrat, countri, gun, gop, fight	6.7
Topic 10	Marriage equality	marriag, gay, contracept, interraci, come, set, alito, rule, stop, argu	6.7
Topic 6	Women’s health access	women, access, care, support, contracept, health, reproduct, free, affect, educ	5.9
Topic 9	Emergency contraceptive Surge	pill, planb, morn, overturn, limit, emerg, demand, purchas, contracept, matter	5.7
Topic 1	Political influence	law, constitut, conserv, major, decid, base, lie, mention, judg, target	5.0
Topic 7	Bodily autonomy	pregnanc, woman, bodi, life, choic, prevent, iud, implant, remov, time	5.0
Topic 14	Physician consultations	day, overturn, plan, talk, doctor, contracept, call, week, increas, news	4.6
Topic 11	Discounts on emergency contraceptives	peopl, understand, pay, stock, lot, friend, happen, offer, overturn, list	3.7
Topic 15	Scaremongering	decis, scotus, contracept, overturn, buy, fear, overrul, limit	3.6

To examine the relationships among topics, we constructed a topic co-occurrence network. In this network, topics identified within the corpus are represented as nodes on a graph. The size of each node corresponds to the frequency of that topic’s appearance in the corpus, with larger nodes indicating higher frequency and smaller nodes indicating lower frequency. The distance between nodes reflects the strength of their connection: nodes positioned closer together are more strongly connected, while those farther apart are less connected. Connections between topics are depicted as edges (lines), where the width of each edge represents the number of co-occurrences observed.

**Fig. 9:**
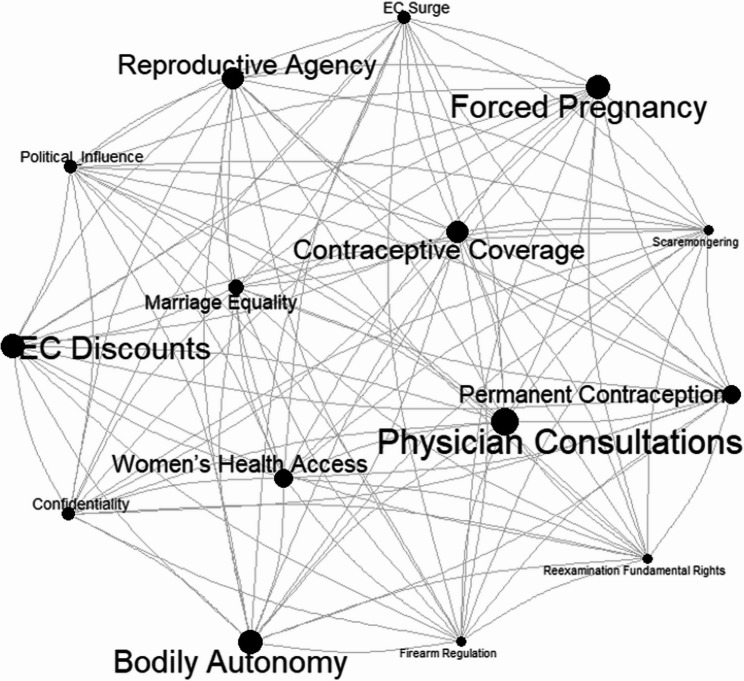
Topic co-occurrence network from the STM illustrating the relationships among 15 topics in the corpus. Each node represents a topic, with the node size proportional to the frequency of the topic’s appearance. Nodes positioned closer together indicate stronger connections, while those farther apart reflect weaker connections. Edges (lines) represent co-occurrences between topics, with edge thickness corresponding to the frequency of observed connections. The network consists of 15 interconnected topics and 400 edges

Figure 9 presents the topic co-occurrence network illustrating how themes identified through topic modeling cluster together within the broader public discourse. Larger nodes represent more frequently occurring topics, while darker and thicker edges indicate stronger co-occurrence between topics within the same tweets. Several noteworthy patterns emerge. First, Physician Consultations, Contraceptive Coverage and Forced Pregnancy appear as central hubs, suggesting that conversations linking clinical guidance, insurance coverage and perceived threats to reproductive freedom were strongly interconnected in the post-*Roe* discourse. Second, topics related to reproductive autonomy, such as Bodily Autonomy, Reproductive Agency and Permanent Contraception, cluster closely with Forced Pregnancy, reflecting concerns about coercion, restricted choice and the erosion of personal decision-making power. Third, Emergency Contraceptive (EC) Discounts and EC Surge show dense linkages with access-related topics such as Women’s Health Access and Confidentiality, indicating heightened public attention to the availability, affordability and privacy considerations surrounding emergency contraception. Taken together, these clusters highlight how practical access issues, perceived threats to reproductive freedom, and clinical decision-making formed an interconnected narrative structure of online discourse following the *Dobbs* decision.

**Fig. 10:**
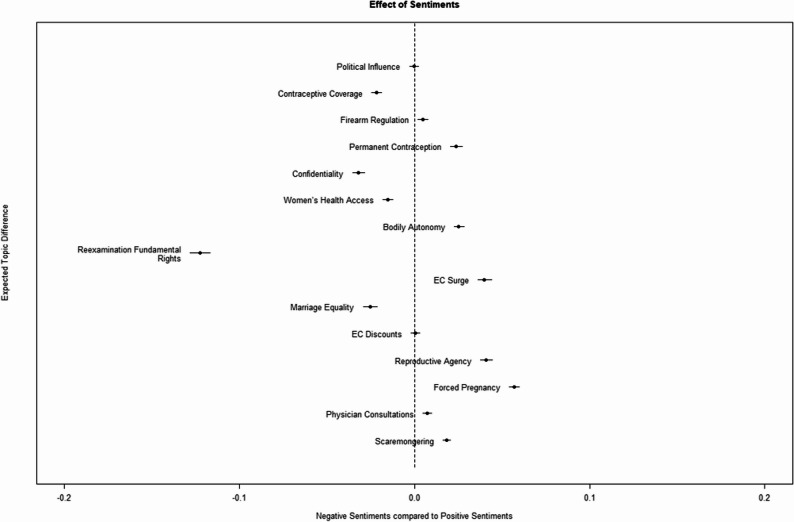
Difference in topic prevalence by sentiments. The x-axis represents prevalence while topics are distributed along the y-axis. Topic < 0.00 indicates the topic has more prevalent negative sentiment while topic > 0.00 indicates the topic has more prevalent negative sentiments. Dots denote point estimates for the difference between topic prevalence for both groups. The lines which bracket the dots represent the 99% Confidence Interval (CI) of that difference. A CI that includes 0.00 is not statistically significant

To understand the emotional valence of tweets within each topic, we examined the sentiment polarity (positive vs. negative) of the 15 topics and compared topic prevalence between these sentiment categories. Figure 10 shows that the topic related to the reexamination of fundamental rights has a particularly strong prevalence in negative sentiments. Other topics with strong negative sentiment prevalence included contraceptive coverage, confidentiality, women’s health access and marriage equality. Conversely, the topic most strongly associated with positive sentiments was forced pregnancy. Other topics with significant positive sentiment prevalence included permanent contraception, bodily autonomy, emergency contraceptive use, reproductive agency, perspectives on implants, firearm regulation and scaremongering. Of the 15 topics analyzed, all except those related to political influence and discounts on emergency contraceptives demonstrated statistically significant differences in sentiment prevalence.

## Discussion

Although previous research has highlighted the importance of analyzing social media discourse on abortion restrictions to guide advocacy and policy [20, 23], limited attention has been paid to public concerns about how the *Dobbs* decision might affect access to sexual and reproductive health services beyond abortion. To address this gap, we examined contraception-related discussions on Twitter (now X) following the overturn of *Roe v. Wade*. Using automated NLP tools, our analysis of the initial weeks post-*Dobbs* revealed that the overall sentiment of contraception-related tweets was predominantly negative. Emotion analysis indicated that “trust”, “anticipation” and “fear” were the most prominent emotions, while STM identified 15 key themes. These included “reexamination of fundamental rights”, “contraceptive coverage”, “forced pregnancy”, “reproductive agency” and “confidentiality”.

Our findings highlight that social media served as a platform for broader discussions on the implications of the Dobbs decision for contraceptive use and access. While recent research has also analyzed Twitter data to understand public reactions to the *Dobbs* ruling, our study is among the few to focus specifically on contraception-related discourse. The predominance of negative sentiments in our findings likely reflects widespread dissatisfaction among users regarding the Supreme Court’s decision. Valdez et al. [67] similarly found that sentiments expressed in tweets after *Dobbs* were overwhelmingly negative, though their work did not address contraception directly. In contrast, Mane et al. [20] reported a higher proportion of neutral sentiments (66.3%) in family planning–related tweets within the broader abortion discourse. This difference may reflect the narrower scope and more comprehensive search strategy of our study, which targeted contraception explicitly. Taken together, these findings suggest that while abortion-related discussions generate broad dissatisfaction, contraception-focused discourse reveals a more nuanced and diverse public response.

Emotion analysis revealed that trust was the most frequently expressed emotion (approximately 21%), followed by anticipation and fear; disgust, anger and surprise were the least common. The frequent expression of “trust” likely reflects individuals conveying strong convictions and certainties rather than positive faith, potentially signaling deep concern that the *Dobbs* decision could threaten contraceptive access. This interpretation is supported by recent evidence from Kavanaugh et al. [6], which suggests significant barriers to obtaining preferred, quality contraceptive care have emerged for women in several states following the overturn of *Roe v. Wade*.

The themes identified through topic modeling underscore the diverse concerns shared by Twitter users. The “reexamination of fundamental rights” was especially prevalent, fueled by concerns that other privacy-based rights might be threatened. These fears were heightened by Justice Clarence Thomas’s concurring opinion, which suggested the Supreme Court should revisit precedents like *Griswold v. Connecticut* (contraception), *Lawrence v. Texas* (same-sex relationships) and *Obergefell v. Hodges* (same-sex marriage) [4, 68]. Such statements intensified public anxieties about future legal actions affecting a broader range of sexual and reproductive rights. Other discussions linked abortion restrictions to contraception more directly. Many users highlighted contraception as a cornerstone of reproductive freedom, essential for preventing unplanned pregnancies and reducing reliance on abortion. However, restrictive abortion laws may inadvertently constrain contraceptive access, further limiting women’s ability to control their reproductive health [1].

The *Dobbs* ruling also brought issues of insurance coverage for contraceptives to the forefront. Users debated the availability and affordability of long-acting methods and questioned the scope and adequacy of current insurance policies. These discussions highlight the urgent need to expand contraceptive coverage in a post-Roe era where abortion access is severely constrained. Furthermore, significant concerns were raised about privacy rights (Topic 5), with users fearing the ruling could set a precedent for revisiting other landmark decisions, highlighting the broader implications of Dobbs for established rights. The co-occurrence of themes like ‘reexamination of fundamental rights’, ‘confidentiality’ and ‘contraceptive coverage’ reveals a public that perceives the Dobbs decision not as a single-issue ruling, but as a systemic threat. The discourse maps a cascade of concerns: from the highest-level legal principles (Topic 8), down to the practical mechanisms of access (Topic 2), and finally to the most intimate scenarios of forced pregnancy (Topic 13).

Psychological Reactance Theory (PRT) provides a useful lens for understanding the individual-level responses to the systemic threats posed by the Dobbs decision. By overturning *Roe v. Wade*, the ruling was widely perceived as a profound “freedom threat”, challenging not only the right to abortion but also the broader framework of reproductive autonomy. Our findings suggest that the emotions expressed in social media discourse, particularly “trust” and anticipation”, as well as the surge in discussions surrounding permanent contraceptive methods, reflect the public’s motivational drive to reclaim agency over their reproductive decisions. In essence, these emotional and behavioral signals illustrate the psychological reactance triggered when individuals perceive external attempts to constrain their autonomy. The heightened uncertainty surrounding access to contraception in the post-*Roe* era appears to further amplify this reactance, prompting individuals to actively seek diverse strategies to protect their reproductive health and maintain control over their fertility. This observed pattern underscores the critical need for targeted policy and advocacy initiatives designed to safeguard women’s reproductive rights. Efforts to ensure equitable access to contraception, expand comprehensive reproductive health education, and dismantle systemic barriers are essential to mitigate the adverse consequences of restrictive abortion laws.

The concerns captured in our data have proven to be prescient, as the post-Dobbs landscape in the U.S. continues to evolve in ways that directly reflect the themes of our analysis. For instance, the prevalent fear of a “reexamination of fundamental rights” is being realized in ongoing legal challenges, such as the Supreme Court case regarding access to mifepristone, which questions the regulatory foundation of medication abortion [69]. Furthermore, the theme of ‘forced pregnancy’ has moved from discourse to reality with the reinstatement of a near-total 19th-century abortion ban in Arizona, which lacks exceptions for rape or incest [70]. These contemporary developments validate the anxieties expressed in our corpus and underscore the importance of the policy recommendations outlined herein, particularly those aimed at protecting contraceptive coverage and safeguarding the right to privacy against continued legal challenges.

### Implications

The findings of this study carry important implications for public health messaging, policy, and clinical practice. The coexistence of high fear alongside assertive certainty in social media discourse indicates that public communications cannot be one-dimensional. Messaging must simultaneously acknowledge and validate the public’s anxieties while providing clear, accurate and actionable information about contraceptive access. It is essential that communications combat misinformation, clarify misconceptions and offer practical guidance, all while addressing the trauma and uncertainty that individuals may be experiencing in the post-Roe era.

Policy responses must similarly reflect this complexity. The pervasive anxiety regarding contraceptive coverage and the apparent surge in demand for methods such as emergency contraception or permanent sterilization highlight the potential for crises in access. Policymakers must act decisively to protect existing protections, such as the ACA’s contraceptive mandate, expand coverage for over-the-counter methods, and ensure that the provision of contraception remains insulated from political and legal challenges. Such policy measures are crucial not only to maintain access but also to prevent further erosion of reproductive autonomy in a landscape reshaped by legal uncertainty.

The evolving discourse also predicts a shifting clinical reality. Patients may present with heightened anxiety, seek permanent sterilization more readily, or express questions grounded in widespread misinformation. Healthcare systems and clinicians must be prepared to respond with trauma-informed, patient-centered care. Providers should be equipped to navigate complex conversations about contraceptive choice under pressure, ensure that all interventions, including sterilization, are delivered with voluntary, informed consent and address patients’ emotional and informational needs comprehensively.

### Limitations

Several limitations should be noted. First, as with all social media data, our findings are subject to biases inherent to unsolicited public posting. Our corpus likely over-represents the views of more vocal and politically engaged individuals, potentially amplifying polarized or extreme viewpoints and under-representing moderate or ambivalent perspectives. Furthermore, as Twitter users are demographically distinct from the general U.S. population, skewing younger, more urban and more liberal, the themes and sentiments we identified may not be generalizable to all Americans. Therefore, our results should be interpreted as a rich analysis of the public discourse within a specific online platform, rather than a direct measure of national public opinion.

Secondly, it is possible that varied and non-conventional terms may have been used to refer to contraceptives/contraception, hence the search terms used to extract the analytic sample of Tweets from the initial dataset may not have captured all tweets related to contraceptive discourse. Additionally, keywords directly related to the *Dobbs* decision (for example “*Dobbs*”, “*Dobbs v. Jackson*”, #AbortionBan) were not used to retrieve the initial data which could have increased the size of the analytic sample of Tweets related to the discourse on contraception. Also, given the racial and ethnic diversity of the US population, Tweets in languages other than English could have provided further insight into discourse. Nevertheless, our findings suggest that further work in this area is warranted.

While tools like the NRC Lexicon for sentiment analysis and STM are powerful for identifying patterns across large datasets, they may lack the nuanced understanding of a human coder. Thus, while our findings represent a high-level, computational mapping of the discourse that captures broad patterns, it may not fully reflect the nuances and contradictions a qualitative analysis would reveal.

Finally, by focusing on a single platform, we acknowledge that our study does not capture the potentially distinct conversations happening on other popular but understudied platforms like Reddit, TikTok or private messaging apps, which may involve different demographics and communicative norms.

## Conclusion

By analyzing the distinct emotional and thematic landscape of contraception discourse post-Roe, this study moves beyond broad public sentiment to uncover the specific anxieties and rhetorical strategies employed by the public. It demonstrates the utility of social media analysis not just for tracking opinion, but for understanding the public’s conceptual mapping of legal threats to their fundamental rights.

## Data Availability

The datasets used and analyzed during the current study are available upon request.
